# Psychiatric care utilization among older people with intellectual disability in comparison with the general population: a register study

**DOI:** 10.1186/s12888-016-1094-0

**Published:** 2016-11-09

**Authors:** A. Axmon, P. Björne, L. Nylander, G. Ahlström

**Affiliations:** 1 Department of Occupational and Environmental Medicine, Lund University, SE-221 00 Lund, Sweden; 2 Research and Development Unit, City Office, City of Malmö, SE-205 80 Malmö, Sweden; 3 Department of Clinical Sciences/Psychiatry, Lund University, SE-221 00 Lund, Sweden; 4 Gillberg Neuropsychiatry Centre, University of Gothenburg, SE-411 19 Göteborg, Sweden; 5 Department of Health Sciences, Lund University, SE-221 00 Lund, Sweden

**Keywords:** Age differences, Cohort studies, Hospitalization, Learning disabilities, Mental retardation, Sex differences

## Abstract

**Background:**

People with intellectual disability have been found to have higher prevalence of psychiatric disorders than the general population. However, they do not seem to have a corresponding increase in psychiatric care utilization. The aim of the present study was to investigate psychiatric care utilization among older people with intellectual disability.

**Methods:**

We used a cohort of people with intellectual disability, 55+ years in 2012 (*n* = 7936), and an equally sized age and sex matched reference cohort from the general population. Psychiatric care utilization was measured using registrations in the Swedish National Patient register during 2002–2012, where each registration corresponds to a psychiatric care occasion.

**Results:**

About 20 % of those with intellectual disability had at least one registration during the study period, compared to some 6 % in the general population sample. In the whole cohort as well as stratified by sex, people with intellectual disability were 3–4 times more likely than those in the general population sample to have had at least one registration during the study period. The effect was, however, only consistent in age groups comprising people younger than 65 years. Among people with intellectual disability, men were more likely than women to have had at least one registration, and people living in special housing (group home or service home) during the entire study period were less likely than those who only lived in special housing for parts of the study or not at all. People with intellectual disability had longer stays per inpatient registration compared with the general population sample. When stratifying on sex, the effect was found only among men, although there were no sex differences within the cohort of people with intellectual disability. Among people with intellectual disability, living in special housing during the entire study period was associated with shorter stays per inpatient registration.

**Conclusions:**

Although people with intellectual disability had higher psychiatric care utilization than the general population during the 11 year study period, it does not correspond to the high prevalence of psychiatric disorders in this population. Future research is required to establish if the level of care utilization is appropriate among older people with intellectual disability.

## Background

People with intellectual disability (ID) have a higher risk for psychiatric disorders than the general population [1, 2]. Thus, in a setting where people with ID have access to health care on the same terms as the general population, a higher psychiatric care utilization would be expected. Only a few studies so far have investigated if it is actually so. Gustafsson [3] investigated psychiatric care utilization in Sweden in the late 1980ies and found that among people with psychiatric disorders, those with co-occurring ID had a lower frequency of overall psychiatric care utilization than those in the general population. In an American setting, Slayter [4] found that among people with substance abuse and co-existing serious mental illness, those with ID were less likely than those without ID to access treatment. On the other hand, Bhaumik et al. [5] found a considerably higher prevalence of psychiatric care among people with ID than in the UK general population. It should, however, be noted that a general population sample was not included in the study, and that the comparison was made using previously published data.

There are indications that prevalence of psychiatric disorders [6] as well as psychiatric care utilization [5] is associated with age among people with ID. Considering this together with the increasing life expectancy for people with ID [7–9], it is important to investigate psychiatric health care needs and utilization among older people with ID. Bhaumik et al. [5] found that people less than 30 years old were more likely than those older to have seen a psychiatrist. However, to the best of our knowledge, no study so far has focused on psychiatric care utilization among older people with ID.

Also in the general population health care needs increase with age. Previous studies have suggested that the relative health care utilization among people with ID compared with the general population is age-dependent [10, 11]. A lower use of psychiatric care among older people with ID compared with the general population could be an indication of health care being based on knowledge about treatment and care of age-related disorders in non-ID populations, which may affect accessibility for people with ID. Therefore, it is important to examine if people with ID have equal access to care in all age groups.

Women with ID have a higher prevalence of psychiatric disorders than men [12], and are also more likely to have a serious psychiatric disorder [13]. However, even though older women with ID have higher psychiatric health care cost than their male counterparts [14, 15], the opposite pattern is found among younger people with ID [15]. In order to ensure equality in access to health care, not only between people with ID and the general population, but also within the group of people with ID, it is important to investigate possible gender differences in psychiatric care utilization among people with ID.

Living arrangements differ between younger and older people with ID, as older people are less likely to have parents who are alive and well enough to care for their adult child. For those who cannot live by themselves, residential care facilities are available in most countries. Such facilities as a rule have around-the-clock staff, and the residents may therefore be expected to be monitored regularly with respect to their health status. If this has any effect on health care utilization, and if so, in which direction, is yet to be determined. Some studies have found people living in group residence to have higher frequency of preventive health care compared with those living with their parents or friends [16, 17]. Whether this has any impact on need for and access to psychiatric care is unknown.

The aim of the present study was to investigate psychiatric inpatient and outpatient care utilization among older people with ID in comparison to the general population. Secondary aims were to investigate age related patterns in psychiatric care utilization among people with ID in comparison to the general population, as well as potential gender differences and effects of living in special housing among people with ID.

## Methods

In this register based study, registers were used both to identify study populations, and to provide information on study outcomes.

### Setting

The Act Concerning Support and Service for People with Certain Functional Impairments (the LSS act) [18] regulates services for three groups of people with disabilities. Group 1 comprises people “with ID, autism or a condition resembling autism” (the two latter henceforth referred to as autism spectrum disorders, ASD) since childhood. Intellectual and physical disabilities occurring later in life, e.g. after a trauma, are categorized as either group 2 or group 3. The LSS act was passed in 1993, and came in place to ensure that people with disabilities have the possibility to shape their lives as they wish. This is done by means of ten specified measures for support and services, such as daytime activities and personal assistance. In order to receive support, the individual applies to the municipality, where a decision is made regarding which group the person belongs to, and the level of support needed. All services provided according to the LSS act are recorded in the LSS register, which is kept at the Swedish National Board of Health and Welfare.

One type of service available for people with disabilities is supported living in a group home or a service home, henceforth referred to as “special housing”. In both types of special housing, each resident has his or her own apartment and access to common areas. Group homes are intended for people with higher need of care, and here the common areas are in direct connection to the apartments. These homes are staffed around-the-clock. In service homes, common areas are in the vicinity of the apartment, but not necessarily in connection to it. Service staff is available at all hours, but not always on site.

### Study cohorts

Through the LSS register, we identified all people in Sweden who fulfilled the following criteria. 1) They were 55 years or older in 2012, 2) they belonged to group 1 (i.e. with ID or ASD), 3) they had received at least one service during 2012, and 4) they were alive at the end of 2012. These people made up the ID cohort (*n* = 7936). As outcome data were obtained for the time period 2002–2012, information was collected for people aged 44 years and above. By using a lower cut-off for “old” than the standard definition, which is 65 years, we hoped to capture the effects of the earlier ageing among people with ID [19].

In order to assess a possible association between psychiatric care utilization and living in special housing, we used information from the LSS register to identify those people in the ID cohort who were living in special housing during the entire study period (*n* = 4661) as well as those who did not, i.e. those who never lived in special housing and those who only lived in special housing during parts of the study period (*n* = 3269).

An age and sex matched sample from the general population was obtained from the Swedish Population register (gPop cohort; *n* = 7936). Each cohort comprised 3609 (45 %) women and 4327 (55 %) men. The mean age on January 1^st^, 2002 (the start of the retrospective data collection) was 53 years (range 44–85) and on December 31^st^, 2012 (at inclusion in the study) it was 64 years (55–96).

### Outcomes

Data from the Swedish National Patient Register was obtained for 2002–2012. This register contains information on inpatient and outpatient visits as described in the Health and Medical Service Act [20]. It does not contain information about visits to primary care. For each visit, the type of clinic is registered based on codes determined by the Swedish National Board of Health and Welfare. One primary and up to 21 secondary diagnoses are registered using the 10th revision of the International Statistical Classification of Diseases and Related Health Problems (ICD-10). In ICD-10, the term “mental retardation” is used rather than “intellectual disability”. However, as “intellectual disability” is the currently preferred term [21], we will use this henceforth.

The outcomes investigated in the present study were psychiatric care utilization. All people in each cohort was classified as having at least one registration in the National Patient register during 2002–2012 or having no registrations during this period. Registrations were considered in total as well as for inpatient and outpatient psychiatric care separately. Moreover, each person’s total number of registrations during 2002–2012 was obtained from the register data. Again, this was done for all types of registrations as well as separately for inpatient and outpatient registrations. Finally, length of stay (LOS) for inpatient registrations was assessed both as the individual’s mean number of days per registration and the individual’s sum of days during the study period.

Considering the age group under study, registrations from “psychiatric care for children and adolescents” were assumed to result from clerical errors and were excluded from the analyses (*n* = 54). Also, registrations were excluded if they did not contain a specification of type of clinic (*n* = 565), as were multiple registrations for the same person, clinic, and day (*n* = 74). Thus, registrations were included if the type of clinic was “general adult psychiatric service”, “psychiatric nursing home”, “geropsychiatric service”, “forensic psychiatric care on regional level”, “specialized psychiatric care”, “alcohol dependency care”, “toxicomania care”, “substance dependency treatment”, or “psychiatric rehabilitation”.

### Statistics

The number of people with at least one registration (inpatient, outpatient, and total, respectively) in the two cohorts were compared by means of odds ratios (ORs) with 95 % confidence intervals (CIs) estimated using logistic regression. Comparisons of number of registrations and LOS, were performed using Mann-Whitney’s *U*-test, as data were skewed.

Analyses were performed for the whole cohorts as well as stratified by sex and age-group (< 59 years, 60–64 years, 65–69 years, 70–74 years, and 75+ years). Also, sex effects and the effect of living in special housing was investigated within the ID cohort.

Inclusion of psychiatric care for the ID itself may inflate the care utilization for the ID cohort in comparison to the gPop cohort. Therefore, we performed subgroup analyses after exclusion of registrations where the primary diagnosis was ID (F7 in ICD-10).

*P*-values less than 0.05 were considered statistically significant.

## Results

### Psychiatric care utilization in the ID cohort

During the study period, there were a total of 10,632 registrations for 1558 people in the ID cohort. Of these, 6539 registrations for 1075 people had another primary diagnosis than ID (F7 in ICD-10). Inpatient registrations accounted for 19 % (22 % when excluding F7-diagnosis). Of people with at least one registration, 403 (26 %) had only one registration, 208 (13 %) had two registrations, 128 (8 %) had three registrations, 125 (8 %) had four registrations, and 694 (45 %) had five or more registrations. The 10 % (*n* = 151) with the highest number of registrations accounted for 40 % (*n* = 4225) of the total number of registrations.

Among the registrations with an F7 diagnosis as primary diagnosis, 28 % had F70 (mild ID), 20 % had F71 (moderate ID), 6 % had F72 (severe ID), 2 % had F73 (profound ID), 1 % had F78 (other ID), and 43 % had F79 (unspecified ID) as primary diagnosis. Subgroup analyses were performed excluding all registrations with an F7 diagnosis.

Excluding diagnoses for ID, the most common primary diagnosis (ICD-10) for inpatient care in the ID cohort was mental and behavioral disorders due to use of alcohol (F10), which accounted for 18 % (*n* = 264), attributed to 47 people, of all registrations, followed by other anxiety disorders (F41, 191 registrations [13 %] for 60 people), pervasive developmental disorders (F84, 152 registrations [11 %] for 65 people), bipolar affective disorder (F31, 147 registrations [10 %] for 46 people) and depressive episode (F32, 123 registrations [9 %] for 66 people).

For outpatient registrations, the five most common primary diagnoses were pervasive developmental disorders (F84, 966 registrations [19 %] for 218 people), bipolar affective disorder (F31, 732 registrations [14 %] for 110 people), other anxiety disorders (F41, 571 registrations [11 %] for 149 people), schizophrenia (F20, 469 registrations [9 %] for 100 people), and depressive episode (F32, 417 registrations [8 %] for 151 people).

Including primary and secondary diagnoses in psychiatric as well as somatic care, 2565 people in the ID cohort had at least one registration with an F7 or Q90 (Down syndrome) diagnosis during the study period, and 398 had at least one registration with an F84 (pervasive developmental disorder) diagnosis. The overlap was 214 people, i.e. 8 % of those with an F7 or Q90 diagnosis also had an F84 diagnosis, and 54 % of those with an F84 diagnosis also had an F7 or Q90 diagnosis.

### Cohort comparisons

People in the ID cohort were 3–4 times more likely than those in the gPop cohort to have at least one registration in total, as well as one inpatient or one outpatient registration (Table 1). When excluding registrations for which the primary diagnosis was ID (F7 in ICD-10), the excess risk of having at least one registration was lowered, but still increased in comparison to the gPop cohort. Among those with at least one registration, the two cohorts did not differ in number of registrations per person when analyzing all registrations (Table 2). However, when excluding registrations with ID (F7) as primary diagnosis, the median number of registrations was higher in the gPop cohort than in the ID cohort. People in the ID cohort had longer LOS per registration compared with the gPop cohort, but no statistically significant difference was found for total LOS during the study period.

**Table 1:** Number of people with at least one registration in the National Patient Register 2002–2012

	All registrations			Registrations with F7 as primary diagnosis excluded		
	gPop	ID	ID vs gPop	gPop	ID	ID vs gPop
	*n* (%)	*n* (%)	OR (95 % CI)	*n* (%)	*n* (%)	OR (95 % CI)
Inpatient						
All	193 (2.4)	573 (7.2)	3.12 (2.64-3.69)	192 (2.4)	420 (5.3)	2.25 (1.90–2.69)
Women	79 (2.2)	240 (6.7)	3.18 (2.46–4.12)	79 (2.2)	183 (5.1)	2.39 (1.83–3.12)
Men	114 (2.6)	333 (7.7)	3.08 (2.48–3.83)	113 (2.6)	237 (5.5)	2.16 (1.72–2.72)
Outpatient						
All	452 (5.7)	1442 (18.2)	3.68 (3.29–4.11)	452 (5.7)	945 (11.9)	2.24 (1.99–2.52)
Women	210 (5.8)	626 (17.3)	2.40 (2.88–4.00)	210 (5.8)	417 (11.6)	2.11 (1.78–2.51)
Men	242 (5.6)	816 (18.9)	3.92 (3.38–4.56)	242 (5.6)	528 (12.2)	2.35 (2.00–2.75)
Total						
All	505 (6.4)	1558 (19.6)	3.59 (3.23–4.00)	504 (6.4)	1075 (13.5)	2.31 (2.07–2.58)
Women	228 (6.3)	674 (18.7)	3.41 (2.91–3.99)	228 (6.3)	478 (13.2)	2.26 (1.92–2.67)
Men	277 (6.4)	884 (20.4)	3.75 (3.26–4.33)	276 (6.4)	597 (13.8)	2.35 (2.02–2.73)

Percentages are based on the entire cohort, i.e. 7936 people in the gPop and ID cohort, respectively

Number of people with at least one registration during 2002–2012 among people with intellectual disability (ID) and an age and sex matched sample from the general population (gPop). Odds ratios (ORs) with 95 % confidence intervals (95 % CI) are estimated by logistic regression

**Table 2:** Number of registrations in the National Patient Register for those with at least one registration, and length of stay (LOS) for those with at least one inpatient registration

	All registrations			Registrations with F7 as primary diagnosis excluded		
	gPop	ID	ID vs gPop	gPop	ID	ID vs gPop
	Median (5-95 %)	Median (5-95 %)	*p*	Median (5-95 %)	Median (5-95 %)	*p*
Inpatient						
All	2 (1–13)	2 (1–12)	0.71	2 (1-13)	2 (1–12)	0.26
Women	2 (1–11)	2 (1–15)	0.41	2 (1–11)	2 (1–13)	0.76
Men	2 (1–14)	2 (1–11)	0.23	2 (1–14)	1 (1–11)	0.079
Outpatient						
All	3 (1–23)	4 (1–19)	0.52	3 (1–23)	3 (1-18)	0.035
Women	3 (1–24)	4 (1–19)	0.65	3 (1–24)	3 (1–19)	0.16
Men	3 (1–21)	3 (1–18)	0.62	3 (1–21)	3 (1–18)	0.12
Total						
All	3 (1–27)	4 (1–22)	0.35	3 (1–27)	3 (1–21)	0.016
Women	3 (1–28)	4 (1–21)	0.43	3 (1–28)	3 (1–20)	0.12
Men	3 (1–26)	4 (1–22)	0.58	2 (1–26)	3 (1–21)	0.064
LOS per visit^a^						
All	8 (1–45)	12 (1–66)	0.002	8 (1–45)	11 (1-75)	0.003
Women	11 (1–45)	12 (1–56)	0.21	11 (1-45)	13 (1–72)	0.21
Men	7 (1–45)	11 (1–79)	0.003	7 (1–45)	11 (1–96)	0.009
LOS for the study period^b^						
All	21 (1–167)	23 (2-302)	0.062	21 (1-167)	21 (2-286)	0.16
Women	25 (1–176)	26 (2–288)	0.24	25 (1–176)	26 (1–211)	0.31
Men	16 (2–167)	19 (2–328)	0.15	16 (2–167)	18 (2–289)	0.37

Number of registrations per person with at least one registration during 2002–2012, and LOS for those with at least one inhospital registration, among people with intellectual disability (ID) and an age and sex matched sample from the general population (gPop). *p*-values are calculated using the Mann-Whitney *U*-test

^a^Individual average per visit during the study period

^b^Total LOS during the study period

**Fig. 1:**
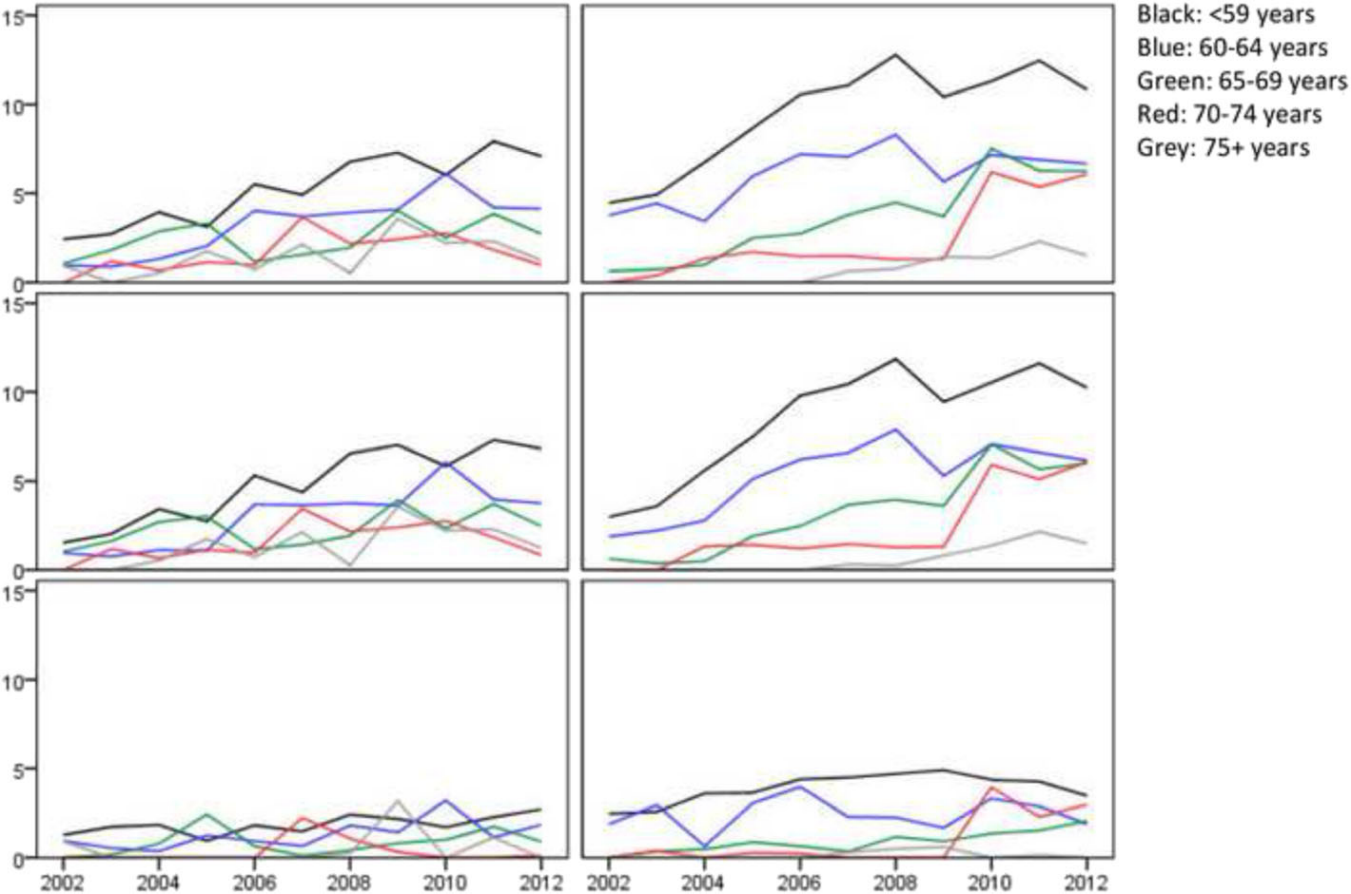
Percentage of people with at least one registration in psychiatric care (in total [*top*], outpatient [*middle*], and inpatient [*bottom*]) in a general population sample (*left*) and among people with Intellectual disability (*right*). Registrations where the primary diagnosis is intellectual disability (F7 in ICD-10) are excluded

**Fig. 2:**
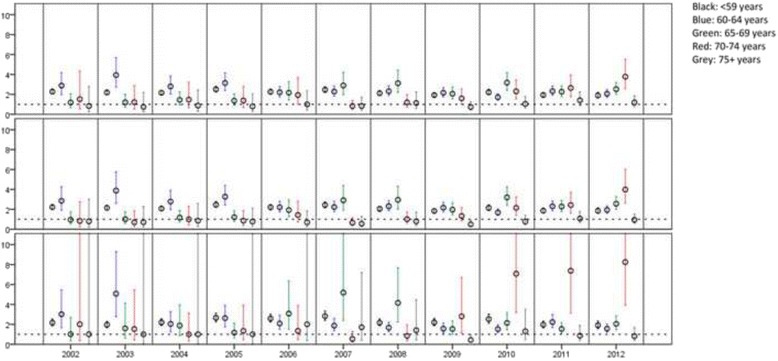
Intellectual disability cohort vs general population sample. Odds ratios for having at least one registration in psychiatric care (in total [*top*], outpatient [*middle*], and inpatient [*bottom*]), with 95 % confidence intervals for comparison between people with intellectual disability, and an age and sex matched sample from the general population. Registrations where the primary diagnosis is intellectual disability (F7 in ICD-10) are excluded

In Fig. 1, the percentage of people with at least one registration is presented by cohort, year, type of registration (inpatient, outpatient, total), and age group. Figure 2 displays risk estimates for the ID vs the gPop cohort per year, type of registration, and age group. Although there are some variations from year to year, the overall pattern for outpatient registrations is an increased OR for people with ID in the younger but not the older age groups. There is also a similar pattern for inpatient registrations, although the smaller sample size caused wider confidence intervals.

### Within cohort effects among people with ID

Within the ID cohort, men were more likely to have at least one registration (OR 1.12, 95 % CI 1.00–1.25), but no statistically significant sex differences were found when analyzing inpatient (1.17, 0.99–1.39) and outpatient (1.11, 0.99–1.24) registrations separately (Table 1). Moreover, when excluding registrations with ID (F7) as primary diagnosis, the gender effect was attenuated and no longer statistically significant (total: OR 1.05, 95 % CI 0.92–1.19, inpatient: 1.09, 0.89–1.32, outpatient: 1.06, 0.93–1.22).

Among those with at least one registration, there were no gender differences with respect to number of inpatient (*p* = 0.098) or outpatient (*p* = 0.22) registrations, or total number of registrations (*p* = 0.15). The results did not change when excluding registrations with F7 as primary diagnosis (*p* = 0.17, *p* = 0.38, and *p* = 0.50, respectively).

LOS did not differ between men and women, neither when assessing the individual average per visit during the study (*p* = 0.39) nor regarding total LOS during the study period (*p* = 0.16). The results were similar when excluding registrations with F7 as primary diagnosis (*p* = 0.33 and *p* = 0.12, respectively).

People with ID living in special housing during the entire study period were less likely to have any type of registration than those living in special housing only part of the study period, or not at all (Table 3). This effect persisted when adjusting for age at the start of the study period. Also, those living in special housing had fewer registrations than those who did not. Among people living in special housing during the entire study period and having at least one hospitalization, the median LOS per visit was 9 days and median LOS for the whole study period was 16 days. For those not living in special housing, or living there only parts of the study period, the corresponding numbers were 13 (*p* = 0.003) and 30 days (*p* < 0.001), respectively.

**Table 3:** Registrations for people with intellectual disability stratified by type of living

	*n* (%)	Median (5-95 %)
Inpatient		
None/some special housing	332 (10.2)	2 (1-14)
Special housing whole study	240 (5.1)	1 (1-8)
None/some vs special housing^a^	OR = 0.48 (0.41–0.57)	*p* = 0.002
- Adjusted for age at study start	OR = 0.50 (0.42–0.60)	
Outpatient		
None/some special housing	656 (20.1)	4 (1-20)
Special housing whole study	784 (16.8)	3 (1-15)
None/some vs special housing^a^	OR = 0.81 (0.72–0.90)	*p* = 0.001
-Adjusted for age at study start	OR = 0.84 (0.75–0.95)	
Total		
None/some special housing	714 (21.8)	4 (1-27)
Special housing whole study	842 (18.1)	3 (1-18)
None/some vs special housing^a^	OR = 0.79 (0.71–0.88)	*p* < 0.001
-Adjusted for age at study start	OR = 0.82 (0.74–0.92)	

Percentages based on number of people in each group, i.e. 3269 in none/some special housing and 4661 in special housing during the whole study period

Number of people with at least one registration during 2002–2012, and number of registrations per person with at least one, among people with intellectual disability

^a^Comparisons between number of people with at least one registration are made using odds ratios (ORs) with 95 % confidence intervals, whereas comparisons between number of registrations per person are compared using Mann-Whitney *U*-test

## Discussion

In the present study, an overall higher utilization of psychiatric care was found among older people with ID compared with the same age group in the general population. This was true for both inpatient and outpatient registrations. However, the difference in psychiatric care utilization between the cohorts was not found among the oldest people in the study. Living in special housing was associated with lower psychiatric care utilization among people with ID.

A strength of the present study is that the outcome measures, i.e. different aspects of psychiatric care utilization, were collected from a national Swedish register to which submission of data is mandatory [22]. Thus, we are likely to have included all inpatient and outpatient registrations for the people in the two cohorts. Also, as it has been suggested that use of psychiatric care varies with age as well as sex [23–25], the use of an age and sex matched reference cohort from the general population is a further strength. However, as no matching beyond age and sex, e.g. by geographical location, was made, we did not have the opportunity to take other potentially relevant factors into account.

The ID cohort is an administrative one, comprising people who have received services according to the LSS act [18]. Since this act was passed in 1993, registration for support is made by application only, and after a systematic assessment made by the social service authority. However, the people in the ID cohort were born before or in 1957, i.e. before the act was passed. At this time, registration for services was more or less automatic for people with an ID diagnosis. Thus, the ID cohort can be expected to fairly well represent the group of ageing people with ID or ASD in Sweden. Nevertheless, that diagnoses are not included in the LSS register may pose a problem. The LSS act regulates services for three groups of people. Only one of these groups, namely those with ID or ASD (group 1), was included in the present study. Within this group, at least one of the listed diagnoses is required to receive support according to the LSS act. However, we cannot tell how many of the people in the ID cohort have a diagnosis of ASD but not of ID. The Swedish National Board of Health and Welfare estimated that among those receiving LSS support during 2004–2010, the group of people with ASD were about half as large as the group of people with ID (including Down Syndrome) [26]. However, the age group investigated in the present study is less likely than younger people to have been diagnosed with ASD. Moreover, a large part of those with ASD also have diagnosis of ID, in the present study as well as in previous studies [27]. Thus, the chance of inflating or disguising the true risk among people with ID is small enough not to affect the generalizability of the results from the present study to other populations with ID.

There is yet another possible weakness associated with the lack of ID diagnoses in that it prevents us from investigating how severity of ID might influence psychiatric care utilization in this large group of older people with ID. Although there is a discrepancy regarding the direction, previous studies suggest associations between severity of ID on one hand and psychiatric disorders and psychiatric care utilization and access on the other [5, 28–31]. Thus, not being able to stratify by severity of ID may cause lower levels of need of psychiatric care in one group to overshadow the higher levels in another, and thereby lead us to underestimate the utilization in the group with the highest occurrence of psychiatric disorders.

Inappropriate, or challenging, behavior such as aggression, antisocial behavior, and self-injurious behavior is common among people with ID [32]. Although not all previous research points in that direction [33], it has been suggested that such behavior may be associated with psychiatric disorders in people with ID [34, 35]. If so, seeking psychiatric care based on challenging behavior may be appropriate. However, if people with ID consult psychiatric care because their caretaker cannot handle their challenging behavior and not due to an actual psychiatric disorder, this would inflate the psychiatric care utilization for people with ID compared with the general population. In ICD-10, conduct disorder is listed as a diagnosis (F91), but this is generally only used for children, and no equivalence is available for adults, much less for older people. Thus, we have not been able to investigate to what extent people with ID are registered in psychiatric care due to no other reason than a challenging behavior.

We found more than three times the risk of having at least one registration among people with ID in comparison to the general population. When registrations with primary diagnosis ID (F7 in ICD-10) were excluded, the risk was reduced, but still doubled among people with ID compared with the general population. The effect was similar for inpatient and outpatient registrations. Moreover, we found a longer LOS among people with ID than in the general population. These results, indicating higher levels of psychiatric care utilization, are in disagreement with previous studies, where no difference – or even a lower psychiatric care utilization – was found among people with ID in comparison to the general population [3, 4, 10, 36]. Among people with ID, a Swedish study found that 0.9 % were admitted to inpatient psychiatric care during one year, compared to 1 % in the general population [3], and a Canadian study found no difference in LOS between people with and without ID [10]. In studies with further diagnostic restrictions imposed on the cohorts, no differences were found regarding outpatient psychiatric care utilization or LOS among people with psychiatric disorders with or without ID [36], and health care access was lower among people with ID than those without in a subgroup of people with substance abuse and serious mental illness [4]. The discrepancy in results may partly be explained by differences in age distributions. However, this does not hold true in the Canadian study, where results stratified by age reveals a lower number of hospitalizations among people with ID compared with those without in the age groups 45–64 years and 65+ years [10]. Other explanations may be differences in health care systems between countries, and selection of groups based on co-existing psychiatric disorders.

An intuitive explanation for the higher psychiatric care utilization among people with ID is that they have a greater need for such health care due to higher prevalence of psychiatric disorders. However, other possible reasons should be acknowledged. The psychiatric care for older people in Sweden is divided between general psychiatry, geriatrics, and primary care, with no care provider carrying the overall responsibility [37]. As a consequence, older people – although having a similar prevalence of psychiatric disorders as younger people – are less likely to be treated in psychiatric care, suggesting that their need of psychiatric care is not properly met by the health care system [38], or that they have a sufficient support system to ensure their need for psychiatric care does not escalate to the level where primary care is no longer enough. People receiving support according to the LSS act [18], on the other hand, are being more closely monitored and should therefore be more likely to come in contact with the health care system when a need arises. However, the lower psychiatric care utilization among people living in special housing argues against this, as do other studies with indications of people with ID having reduced access to health services [39].

It has been suggested that there is a stigma surrounding psychiatric care causing many to refrain from seeking help even when they need it [40, 41]. If such reluctance is more common in the general population than among people with ID, or people in the general population have a higher degree of freedom to determine whether to seek psychiatric care, this could be another contributing explanation to the difference in psychiatric care utilization found in the present study.

We found a small increased risk for men with ID to have at least one registration in psychiatric care compared with their female counterparts. As women with ID have been found to have a higher prevalence of psychiatric disorders than men [12], and more likely to have a serious psychiatric disorder [13], this could be an indication of a sex difference in the health care barriers already present for people with ID. However, after exclusion of registrations where the primary diagnosis was ID (F7 in ICD-10), the risk was attenuated. This would suggest that men with ID have a higher utilization of psychiatric care for their ID compared with women, but that there are no gender differences with respect to co-existing psychiatric disorders.

Among people 65 years or older, the increased psychiatric care utilization found for younger age-groups was attenuated or non-existent. A similar pattern was found when examining somatic health care in the same cohorts [42], and also in a Norwegian study assessing hospital admissions among people with ID [43]. As already mentioned above, a Canadian study found decreasing hospitalization for psychiatric disorders with increasing age among people with ID, both in absolute numbers and in comparison to the general population [10]. A decrease relative to the general population could occur if the older groups with ID used less health care than the younger groups with ID. It would also be seen if older groups of people in the general population used more health care than the younger, which would be a fair assumption considering the increasing risk for ill-health with increasing age. In the present study, the decrease in excess risk for people with ID associated with older age seems to be driven by a decrease in health care utilization in the oldest age group. As the same pattern is seen for each year during the study period, it is unlikely that the age-related decrease in health care utilization stems from a cohort effect. It is more probable that it is due to a survival selection, i.e. that the people in the older age groups are healthier because the less healthy did not reach this age. This is supported by previous studies where psychiatric disorders among people with ID were found to decline with increasing age [44].

In the 1970ies, the de-institutionalization began in Sweden. Today, no person with ID is institutionalized. Those who cannot live alone or with their family, may apply for special housing. In the present study, people with ID who lived in special housing during the entire study period had lower levels of psychiatric care utilization than those who did not. This is in agreement with a Dutch study, in which people with ID who were living alone had higher odds of being heavy users of mental health care compared with those living in family or residential stay [30].

A small group of people with ID accounted for a large part of the total number of registrations. The same pattern has been found among people with ID with referrals to a community based mental health service in the UK [45]. This skewness in the distribution of registrations could suggest the presence of a group of people with ID and severe, or complex, psychiatric co-existing disorders. Thus, there is a need to investigate predictors for heavy psychiatric care utilization.

## Conclusions

Even though recent studies suggest that the prevalence of psychiatric disorders may be as high as 40 % among people with ID, less than 20 % of the people with ID in the present study used psychiatric care during the 11-year study period. Whether this is an indication of unmet health care needs among people with ID is an urgent question for future research, as is the issue of how primary health care meets the need of preventive efforts and health care in people with ID and psychiatric disorders.

## Abbreviations

ASD: Autism spectrum disorder
CI: Confidence interval
gPop: General population
ICD-10: International statistical classification of diseases and related health problems, 10th revision
ID: Intellectual disability
LOS: Length of stay
LSS: The act concerning support and service for people with certain functional impairments
OR: Odds ratio
